# A flexible wearable self-supporting hybrid supercapacitor device based on hierarchical nickel cobalt sulfide@C electrode

**DOI:** 10.1038/s41598-023-42278-9

**Published:** 2023-09-20

**Authors:** Xin Chen, Ming Sun, Fadi Jaber, Erfan Zal Nezhad, K. S. Hui, Zhenwu Li, Sungchul Bae, Muge Ding

**Affiliations:** 1 Department of Mechanical Engineering, Heze University, Heze, Shandong China; 2 Department of Biomedical Engineering, Ajman University, Ajman, United Arab Emirates; 3 Center of Medical and Bio-Allied Health Sciences Research, Ajman University, Ajman, United Arab Emirates; 4 Department of Biomedical Engineering and Chemical Engineering, University of Texas at San Antonio, San Antonio, TX USA; 5 School of Mathematics, University of East Anglia, Norwich, NR4 7TJ UK; 6 Department of Architectural Engineering, Hanyang University, Seoul, South Korea; 7 Engineering Faculty, Auckland University, Auckland, New Zealand

**Keywords:** Chemistry, Energy science and technology

## Abstract

A flexible wearable electrode consisting of nickel–cobalt sulfide (NCS) nanowires was fabricated in this study. Self-supporting NCS was grown in situ on porous carbon nanofibers without a binder as a novel material for supercapacitor electrodes. The NCS nanowires were grown using cyclic voltammetry electrodeposition, which proved to be a fast and environmentally friendly method with good controllability of the material structure. One-dimensional carbon nanofibers (C) have high surface-area-to-volume ratios, short ion transmission distances, excellent mechanical strengths, and remarkable flexibilities. Moreover, the NCS@C flexible electrode exhibited a synergetic effect with the active compounds, and the dense active sites were uniformly distributed across the entire surface of the carbon fibers, enabling rapid electron transport and enhancing the electrochemical properties of the NCS@C nanowires. The NCS@C achieved specific capacitances of 334.7 and 242.0 mAh g^−1^ at a current density of 2 A g^−1^ and high current densities (up to 40 A g^−1^), respectively, corresponding to a 72.3% retention rate. An NCS@C-nanofilm-based cathode and an activated-carbon-based anode were used to fabricate a flexible asymmetric supercapacitor. The device exhibited high energy and power densities of 12.91 Wh kg^−1^ and 358 W kg^−1^, respectively.

## Introduction

Flexible and wearable electronic devices can be used as foldable capacitive touch screens and curved smartphones in numerous applications, such as motion detection and microrobotics. Intelligent, flexible energy storage, and conversion devices with low weight, high safety, small volume, excellent electrochemical performance, and good mechanical durability are in great demand^1^. Flexible supercapacitors are promising candidates because of their strong mechanical flexibility and high safety even under mechanical distortion. However, they must also have a long cycle life, high energy density, and excellent electrical conductivity^2^. A flexible supercapacitor generally comprises a compatible electrolyte and a separator in a flexible assembly and a flexible electrode with excellent electrochemical characteristics^3^. Carbon materials are widely used as flexible electrode materials, conductive additives, and supporting substrates in electrochemical energy storage devices because of their large surface areas, good electrical conductivities, and outstanding chemical stabilities^4^. Carbon fibers containing > 90% carbon are expected to improve the mechanical properties of flexible supercapacitors owing to their high elastic moduli and appropriate mechanical strength^5^. Carbon fiber textiles have outstanding flexibility but a low surface electrochemical reactivity and small surface area^6^. Nonetheless, the synthesis of hierarchical pores and various pseudocapacitive oxygenic groups transforms carbon fiber textiles into highly active electrodes with adequate cycling durability and high capacitance^7^. Transition metal sulfides with nanoscale structures have attracted broad interest because of their unique advantages in energy storage applications^8^. The electrochemical performance of most cobalt sulfide nanosheet arrays is unsatisfactory owing to the limited number of redox-active sites because their electronic structure is not properly regulated. Partially replacing the electrochemically inactive Co^2+^ ions that control the cobalt sulfide bond with inexpensive, environmentally benign, first-row transition metal cations would be beneficial for achieving a high charge storage capability, ionic conductivity, and extended cycle durability. In particular, nickel, which neighbors cobalt (atomic number 27; ionic radius = 0.79 Å) in the periodic table and has a similar atomic radius (0.83 Å) to Co, would be a superior choice for controlling cobalt sulfide bonds. Adding Ni^2+^ ions to the parent cobalt sulfide bonds results in the Ni^2+^ ions replacing inactive Co^2+^ ions close to the active sites, thus inducing more electrochemically active sites, higher redox activity, and high charge storage capability^9^. Nickel–cobalt sulfide (NCS) exhibits appealing properties for energy storage devices, including low toxicity, low cost, and high performance, making the energy storage devices competitive electrochemical devices^10^. Owing to their narrower bandgaps and higher electrical conductivities, binary metal sulfides are expected to outperform metal oxides in electrochemical devices. Therefore, NCS-based electrode materials with adjustable nanomorphologies, such as nanosheets, nanoparticles, and nanotubes, have been extensively used to mitigate the issues mentioned above. NiCoS exhibits a high specific capacitance (SC) because of its multiple valences^11^. For example, Soram et al. synthesized a flexible and transparent core–shell Cu@Ni@NiCoS nanofiber electrode that exhibited an outstanding high area capacity of 6.94 Ah cm^−2^ and a transmittance of 76.83%^12^. Because NiCoS electrodes are highly dependent on the Faraday redox reaction and ion diffusion during rapid charge–discharge processes, they cannot be used for large-scale practical applications because of their poor rate performance and electrochemical stability. Qiu et al*.* synthesised NCS using single- and two-step hydrothermal methods^13^, and the NCS exhibited a remarkable SC of 242.0 mAh g^−1^ as a pseudosupercapacitor electrode. However, the actual percentage of electroactive materials can decrease significantly when severe aggregation of NCS occurs, resulting in hindered electron transfer and ion diffusion^14^. In this study, a facile two-step process was developed to fabricate novel nanoarray-structured NCS@C electrodes. The conversion of polymer fibers into carbon fibers requires both stabilization and carbonization. In this process, the removal of heteroatoms and volatilization of residual solvents affect the molecular structure, and the fiber diameter decreases owing to thermal shrinkage, forming a larger specific surface area, as compared to that of the original polymer fibers^15^. Optimization of the electrodeposition time using the appropriate NCS precursors can create a nanoarray morphology that increases the ion content in the electrode construct and facilitates electron transfer^16^. The NCS precursor was prepared using a special pulse electrodeposition method on porous carbon fibers, making the nanoarray structure controllable, fast, simple, and suitable for commercial batch production. Optimization of the electrodeposition time revealed that 40 s yielded the best-performing materials with the highest SC (334.7 mAh g^−1^ at a 2 Ag^−1^ current density) and cycling stability after 4,000 cycles (80.8% preserved capacitance), as compared to the 20 and 80 s electrodeposition time. Moreover, a device with an NCS@C cathode and an activated carbon-based anode yielded an energy density of 12.91 Wh kg^−1^ and a power density of 358 W kg^−1^, demonstrating the application of the new materials in a real-world device. These results suggest the strong potential of the electrochemically deposited NCS@C nanowires as competitive candidates for supercapacitor electrodes.

## Experimental

### Synthesis of carbon nanofibers

To prepare the electrospinning precursor solution, polyacrylonitrile (PAN; 3 g) was mixed with N,N-dimethylformamide (22 g, AR, ≥ 95), and the mixture was vigorously stirred at 60 °C until a homogeneous solution was formed (12 w% PAN)^17^. The PAN nanofiber precursor was electrospun from the as-prepared solution onto an aluminum foil collector. A voltage of 18 kV was applied between the aluminum collector and the 20# needle tip, which were 18 cm apart^18^. A constant flow rate of 0.8 ml/h was adopted. The collected PAN nanofiber membrane precursor was baked in a furnace at 260 °C for 4 h in air using a 1 °C min^−1^ heating rate for the pre-oxidization process^19^. The pre-oxidized fiber membrane was baked at 800 °C for 2 h in a tube furnace for carbonization (using a 1 °C min^−1^ heating rate) in the presence of argon gas^20–22^.

### Synthesis of NiCo_2_S_4_ nanoarrays

All chemicals used for the synthesis of NCS were of analytical grade and used without additional purification. An electrodeposition hydrothermal process was used to synthesize NiCo_2_S_4_ nanoarrays with different morphologies^23^. Prior to NCS synthesis, the carbon nanofibers (substrates) were cleaned successively with nitric acid, ethanol, and deionized (DI) water^24^. In the first electrodeposition process, NixCo_1-x_(OH)_2_ with a layered morphology was deposited on the carbon nanofibers^25^. The material was electrodeposited using a standard three-electrode setup (working electrode: Ni_2x_Co_x_(OH)_2_-coated carbon nanofibers, reference electrode: saturated Ag/AgCl, and counter electrode: platinum foil) at room temperature^26^. A metal hydroxide solution (0.1 M, 70 ml) with a Ni^2+^/Co^2+^ mole ratio of 1:2 was used as the electrodeposition electrolyte. Under a − 1.2 to 0.5 V potential, the Co_2x_Ni_x_(OH)_2_ acicular crystals were deposited on the carbon fiber cloth within 40 s. Dynamic potential cyclic scanning was used for the electrodeposition. The potential sweeping cycle was 0 to − 1.2 to 0.5 to 0 V. Samples with different deposition times, namely, 20, 40, and 80 s, were prepared. Cyclic voltammetry (CV) revealed that the sample with the 40 s deposition time exhibited the best electrochemical performance^27^. Next, the precursors were mixed with a Na_2_S solution (35 mL, 0.2 M) in an 80 mL Teflon-lined stainless-steel autoclave. The solution was incubated at 120 °C for 14 h^28^. The NCS@C electrode was removed from the liquid phase and rinsed with DI water and ethanol. The mixture was then maintained at 70 °C for 12 h^29^.

### Assembly of asymmetric supercapacitor (ASC) devices

ASC devices with a two-electrode structure were assembled with the as-prepared NCS nanowires@C (cathode), AC (anode), and a polymer electrolyte separator (polyvinyl alcohol (PVA)-KOH) between them^30^. To prepare the AC electrode, polytetrafluoroethylene, acetylene black, and the active materials were mixed in a 1:1:8 mass ratio. The preparation of the PVA-KOH polymer electrolyte was based on a standard process^31^. First, PVA (6 g) was mixed with distilled water (60 mL) and the mixture was stirred for 2 h at 85 °C. Subsequently, a KOH solution was prepared by dissolving KOH (4 g) in DI water (20 mL). Finally, the PVA and KOH solutions were mixed and continuously stirred at 80 °C until a clear mixture was obtained. The prepared materials were submerged in the electrolyte solution for 8 min. The materials were dried under ambient conditions before the two electrodes were assembled. After the PVA-KOH solidified, the final ASC device was obtained.

### Characterization

X-ray diffractometry (XRD) with Cu-Kα (l = 1.5418 Å) radiation at 40 kV and 40 mA was used to characterize the crystal structure and phase purity of the NCS. All samples were measured at 5–90° in an ambient environment^32^. X-ray photoelectron spectroscopy (XPS) analyses were performed using an ESCA2000 with an Al Kα excitation laser to determine the chemical state of the elements^33^. Field emission scanning electron microscopy (FE-SEM) and high-resolution transmission electron microscopy (HRTEM; JEM-2100F) were used to characterize the morphology of the NCS nanostructures. The Brunauer–Emmett–Teller (BET) surface area and porous properties of NCS@C were examined using nitrogen adsorption/desorption isotherms obtained using a surface area and porosity analyzer (TriStar II 3020, version 3.02, Micromeritics Instrument Corporation).

### Electrochemical measurements

All electrochemical experiments were conducted on an RST 5100F platform using a conventional three-electrode setup^34^. A 3 M KOH solution was used as the electrolyte. The as-fabricated NCS@C electrodes were used as the working electrode, a platinum plate was used as the counter electrode, and a Hg/HgO electrode was used as the reference electrode. In the CV measurements, 0–0.6 V was used as the window for the potential scan^35^. The scan rate was set at 5, 10, 30, 50, 70, and 100 mV s^−1^. The galvanostatic charge–discharge (GCD) process was conducted in the potential range of 0–0.55 V using current densities of 1, 2, 6, and 10 A g^−1^. An ASC device was fabricated using NCS@C and AC@C as the cathode and anode, respectively, and a PVA-KOH gel was used as the electrolyte^32^. The weight of the anode material was calculated by charge-balancing between the two electrodes.

## Results and discussion

### Synthesis and characterization

**Figure 1:**
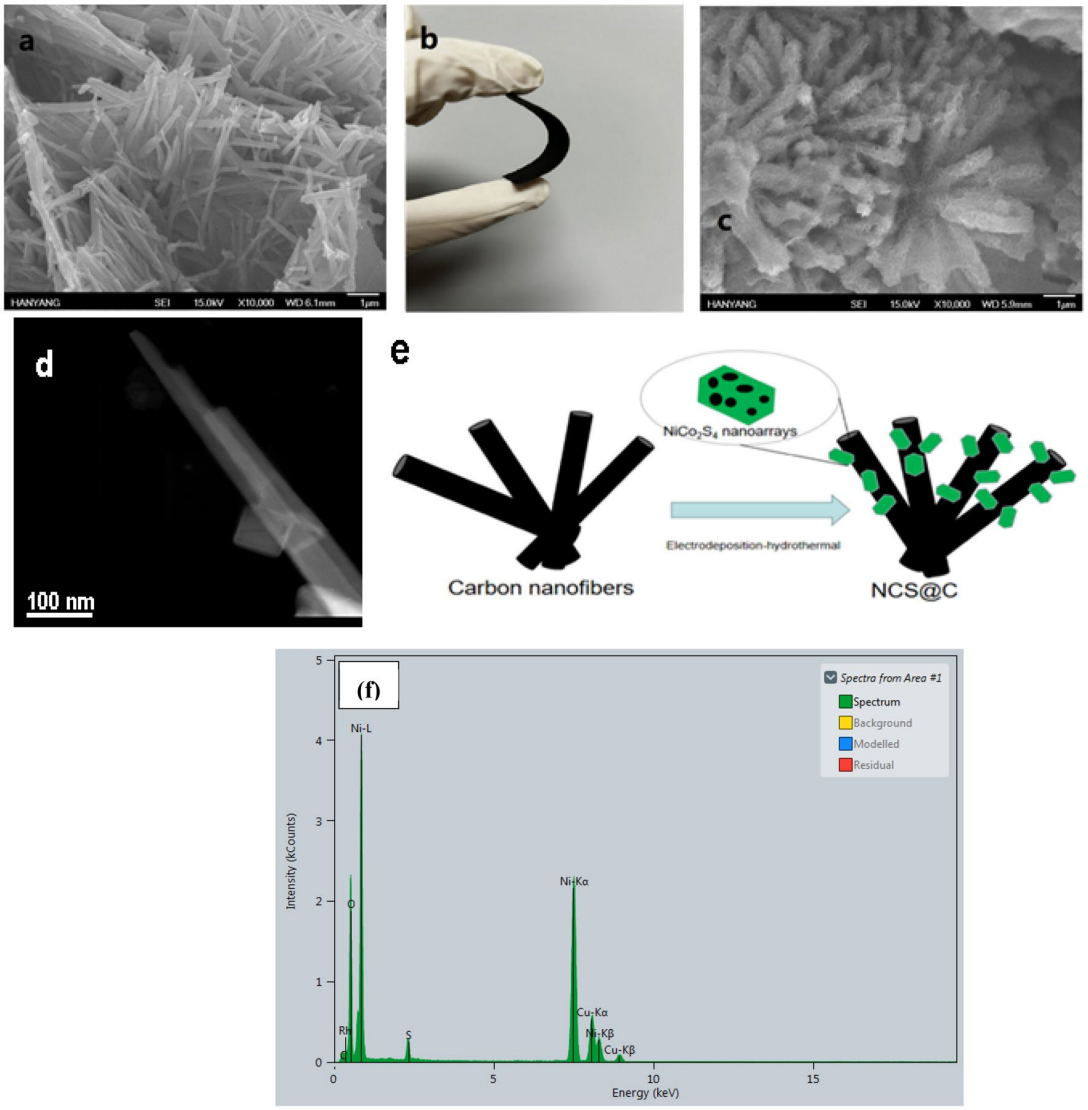
(**a**) SEM image of the carbon nanofibers, (**b**) photographic image of the flexible NCS@C electrode, (**c**) SEM image of NCS@C, (**d**) TEM image of a carbon nanofiber, (**e**) illustration of the NCS@C electrode preparation process, and (**f**) the EDX spectrum of the NiCoS electrode.

Figure 1e illustrates the NCS@C nanowire array synthesis process. Figure 1a–f shows a SEM image of the carbon nanofibers, a photographic image of the flexible NCS@C electrode, a SEM image of NCS@C, a TEM image of the carbon nanofiber, an illustration of the NCS@C electrode preparation process, and the EDX spectrum of the NiCoS electrode, respectively. NCS nanowires are generally grown on carbon fiber substrates via hydrothermal electrodeposition, followed by hydrothermal processes. First, NCOH was directly electrodeposited onto a carbon fiber substrate. The subsequent hydrothermal process yielded an NCS nanowire array. During electrodeposition, the NCOH precursors are typically Ni(II) and Co(III) nitrates, and a cathodic potential is applied to reduce these nitrates and H_2_O. OH^−^ ions (or consumed H^+^) can be produced close to the electrode surface, leading to an increase in the pH and precipitation of NCOH. The mechanisms are shown in Eqs. (1–3)^36^:

1 $$ {\text{NO}}_{3}^{ - } + {\text{H}}_{2} {\text{O}} + 2{\text{e}}^{ - } \to {\text{NO}}_{2}^{ - } + 2{\text{OH}}^{ - } $$

2 $$ {\text{NO}}_{3}^{ - } + 7{\text{H}}_{2} {\text{O}} + 8{\text{e}}^{ - } \to {\text{NH}}_{4}^{ + } + 10{\text{OH}}^{ - } $$

3 $$ x{\text{Ni}}^{2 + } + 2x{\text{Co}}^{2 + } + 6x{\text{OH}}^{ - } \to {\text{Ni}}_{x} {\text{Co}}_{2x} \left( {{\text{OH}}} \right)_{6x} $$

Consequently, Na_2_S·9H_2_O was added to the composite electrode during hydrothermal processing. Na_2_S released reactive sulfide ions (S^2−^) during hydrolysis, which induced the nucleation of NiCo_2_S_4_ crystals owing to anion exchange in the sulfidation step. Nickel and Co ions in NCOH can receive two electrons from the S^2−^ anion and cluster with it after dissociation with OH^−^ (Eq. 4)^37^.

4 $$ {\text{Ni}}_{x} {\text{Co}}_{2x} \left( {{\text{OH}}} \right)_{6x} + 4x{\text{S}}^{2 - } \to x{\text{NiCo}}_{2} {\text{S}}_{4} + 6x{\text{OH}}^{ - } + 2x{\text{e}}^{ - } $$

**Figure 2:**
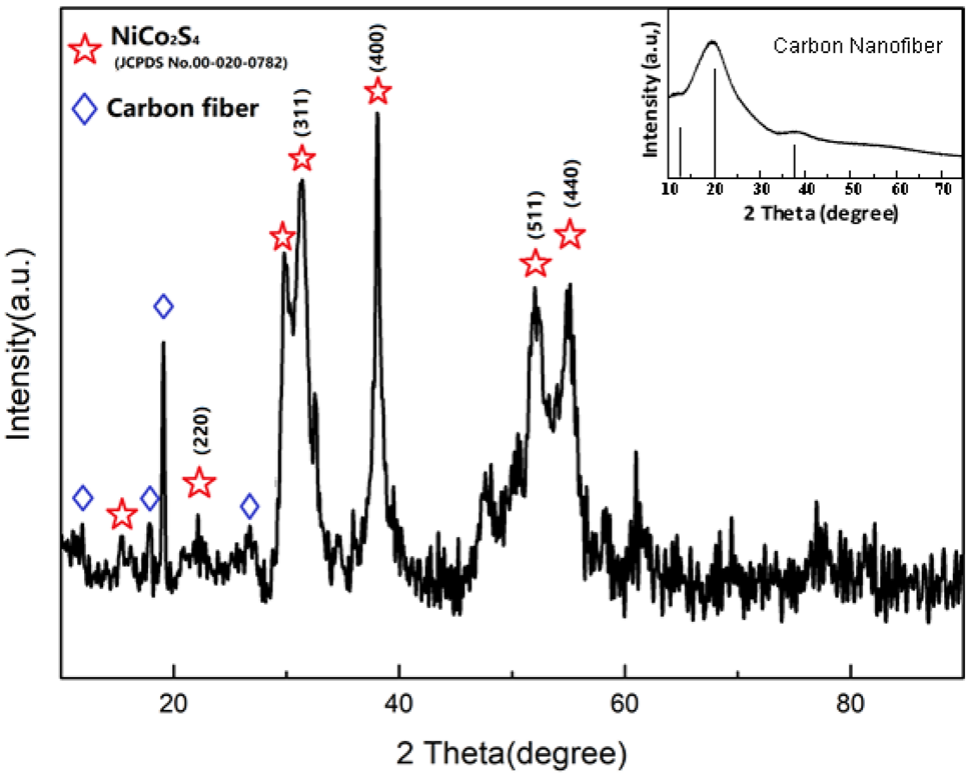
XRD patterns of NCS@C.

The phases and structures of the NCS nanowire arrays were characterized using XRD. As depicted in Fig. 2, the peaks of the NCS NWAs were consistent with those of cubic-phase NiCo_2_S_4_ (JCPDS No. 00-020-0782)^28^. The typical peaks for the (220), (311), (511), and (440) planes of the NCS phase indicate that highly crystalline NCS nanosheets were formed on the carbon fiber. The XRD patterns of the carbon nanofibers are shown in Fig. 2.

**Figure 3:**
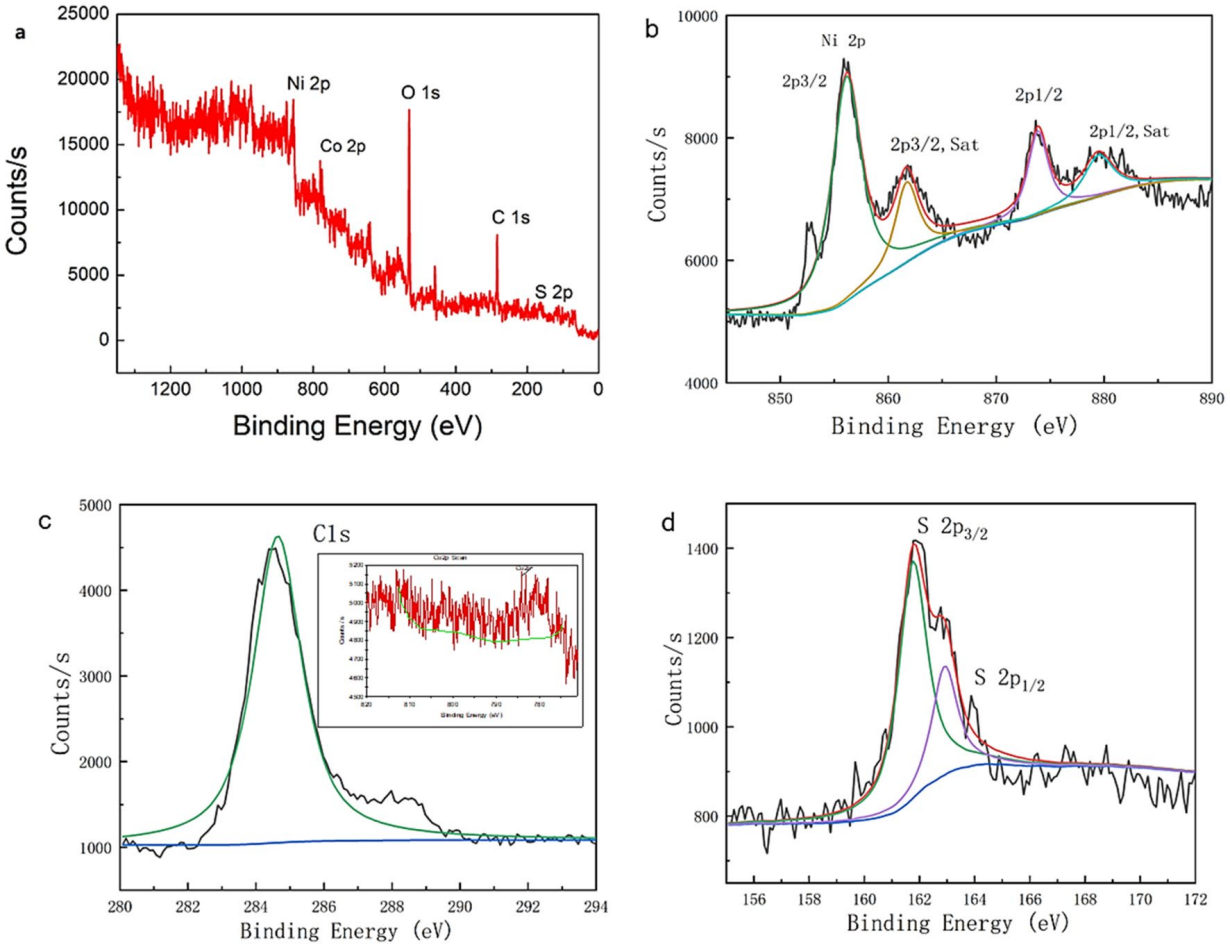
Wide survey of the XPS spectrum of NCS@C. (**a**) High-resolution XPS spectra of (**b**) Ni 2*p*, (**c**) C 2p, and (**d**) S 2*p*.

XPS was used to further examine the chemical compositions and valence states of the active materials. The full XPS spectrum in Fig. 3a shows that the material contained Ni, C, and S. Based on Gaussian fitting, two doublets due to spin–orbit coupling and two shakeup satellite peaks were observed in the original Ni 2*p* spectrum (Fig. 3). The coexisting Ni(II) and Ni(III) were indicated by two strong peaks at 874.3 and 856.0 eV for Ni 2*p*_3/2_ and Ni 2*p*_1/2_, respectively^34^. Figure 3 shows the S 2*p* region of S. The peaks at 160.9 (2*p*_3/2_) and 162.85 eV (2*p*_1/2_) clearly demonstrate the presence of NiCo_2_S_4_ in the product, and no oxide impurities, such as NiCo_2_O_4_, were detected. The XPS analysis showed that the sulfide nanosheets were composed of multivalent transition metal elements that can provide rich redox reaction sites and excellent conductivity.

**Figure 4:**
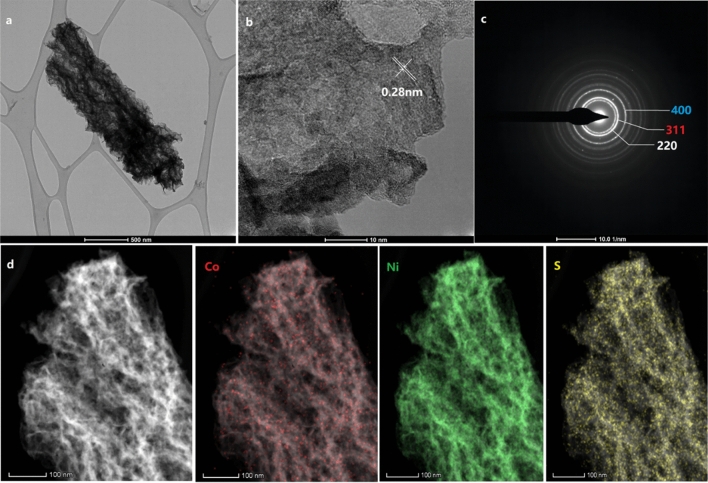
(**a**) TEM, (**b**) HRTEM, and (**c**) SAED images of an individual NCS@C nanosheet and (**d**) the corresponding EDS mapping results.

The capacitance and cycle stability of ASCs are highly dependent on the morphology of the electrode material. The SEM images (Fig. 1a and c) show that the NCS nanosheets uniformly covered the carbon fibers, developing a hierarchical structure, and that the nanosheets were homogeneously wrapped around the nanowires. As shown in Fig. 1a, the obtained carbon nanofiber samples exhibited a smooth surface, relatively uniform diameter distribution, and no bead string structure. Uniform NiCo_2_O_4_ nanowires were successfully grown on the carbon fiber surfaces (Fig. 1c). To comprehensively understand and analyze the morphology- and structure-dependent properties of the NCS@C nanowires, the electrodeposition time was varied (20, 40, and 80 s) using well-controlled solution concentrations and temperatures. A deposition time of 40 s yielded the optimal morphology, and the sample outperformed the others in terms of electrochemical properties. The separated nanowires were supportive; they offered more surface area to the network structure of the carbon fibers and provided channels for ion transport. These orderly nanowire network structures produced numerous open channels and surfaces with dense electroactive sites, which offer more active sites for redox reactions and expose more active material to the electrolyte, leading to an enhanced SC and cycle stability. Different deposition times resulted in different material structures, which can affect conductivity and cause a shift in the redox peaks. The microstructure of NCS@C was examined using HRTEM. Figure 4a shows that nanoflakes were distributed on the surfaces of the carbon fibers. The HRTEM image of a single nanoflake shows clear lattice fringes with a spacing of 0.28 nm (Fig. 4b), which were indexed to the (220) planes of NCS. Figure 4c shows the diffraction rings in the SAED pattern, which matched well with the (220), (311), and (400) planes of the NCS, indicating the presence of NCS@C. Figure 4d shows the EDS elemental mapping used to determine the elemental distribution in the NCS@C sample. Overall, Co, Ni, and S were uniformly distributed on NCS@C.

**Figure 5:**
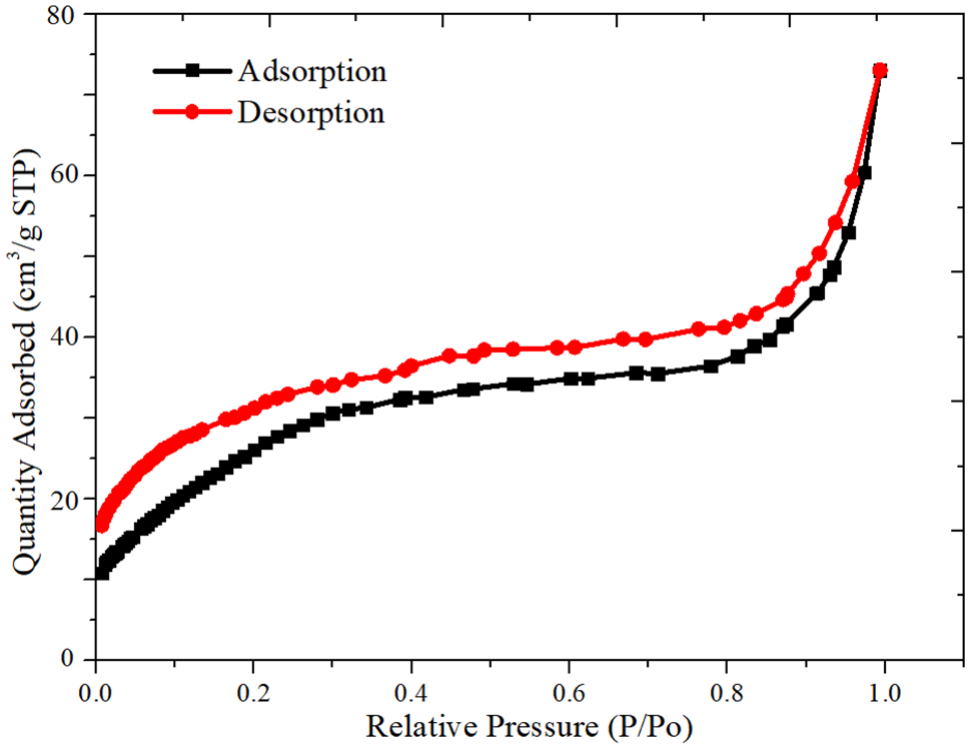
BET analysis results of the nitrogen adsorption and desorption isotherms for the NCS@C.

Nitrogen adsorption/desorption measurements were performed to examine the porosity and BET surface area of the NCS@C. All N_2_ adsorption/desorption isotherms in Fig. 5 were typical type IV isotherms with a hysteresis loop in the P/P0 range of 0.3–0.8, suggesting that the materials had a mesoporous structure. These curves are based on the IUPAC classification of type IV isotherms with loop hysteresis. The single-point surface area at P/Po = 0.2915360 was 9.3382 m^2^/g. The BET and Langmuir surface areas of P/Po were 9.7151 and 16.6330 m^2^/g, respectively. The t-plot micropore area and external surface area were 7.1754 and 2.5397 m^2^/g, respectively. The BJH adsorption–desorption cumulative surface area of pores with a diameter of 1.000–3,000.000 Å were 13.460 and 16.2958 m^2^/g, respectively.

### Electrochemical characterization of electrodes

**Figure 6:**
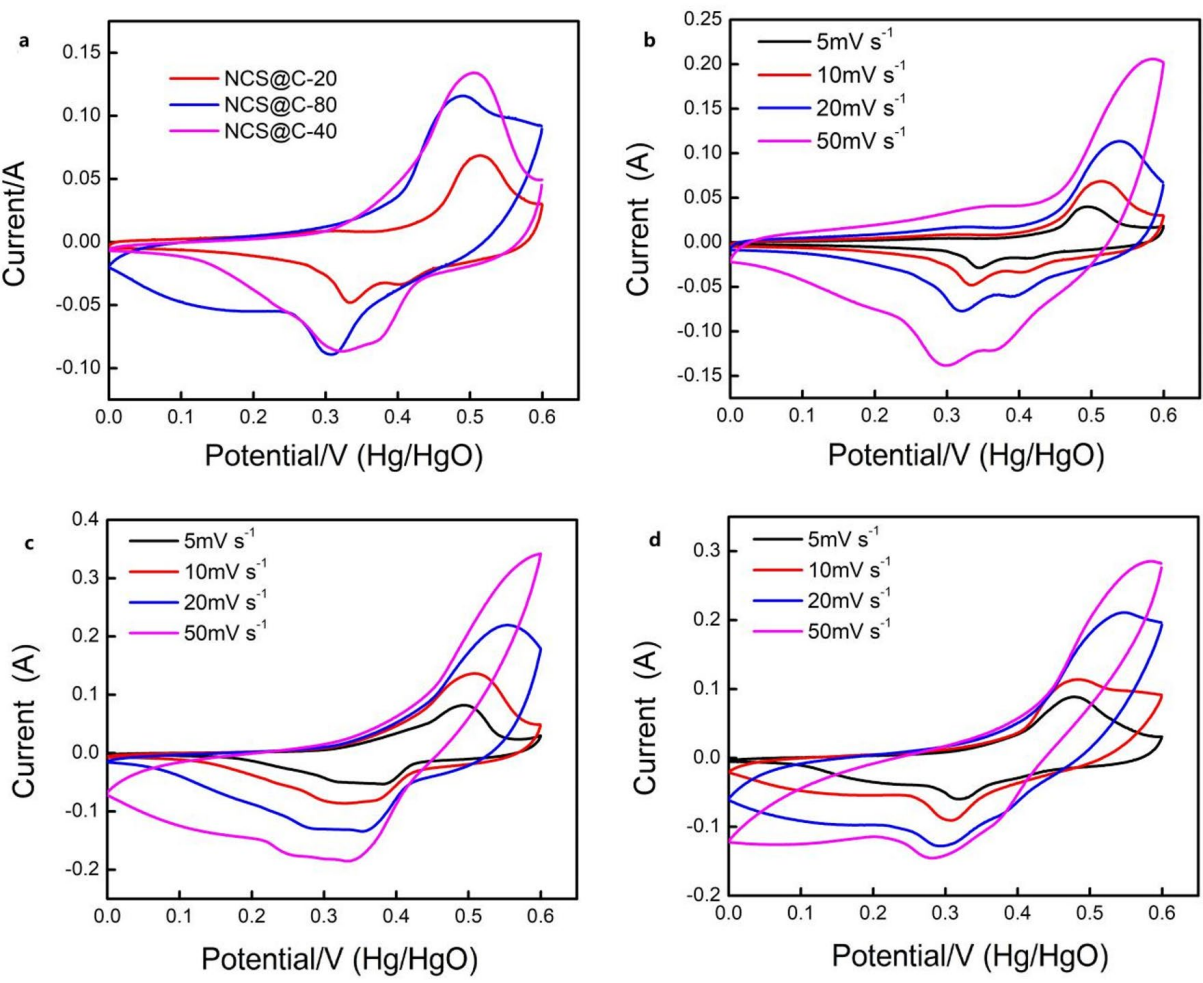
(**a**) CV curves at a scanning rate of 10 mV s^−1^ and (**b**), (**c**), and (**d**) CV scans of the three samples at the scanning rates of 5–50 mV s^−1^.

Three electrochemical methods (electrochemical impedance spectroscopy (EIS), GCD, and CV) were used to characterize the NCS samples^38^. The correlation between the deposition time and the electrochemical properties was explored. The samples with the electrochemical deposition times of 20, 40, and 80 s were labeled as NCS@C-20 (0.6 mg/cm^2^), NCS@C-40 (1.4 mg/cm^2^), and NCS@C-80 (0.78 mg/cm^2^), respectively. CV scans of the NCS@C nanowires at various electrodeposition times and a scanning rate of 10 mV s^−1^ are shown in Fig. 6a. Representative CV scans of the electrodeposited NCS@C electrodes with scanning rates ranging from 5 to 50 mV s^−1^ are shown in Fig. 6b–d. The currents of the four samples increased with an increasing scanning rate. The oxidation and reduction peaks shifted to more negative and positive potentials, respectively. This phenomenon is due to the relatively slow shift of the alkali ions with increasing scanning rates. Because the alkali ion rate shift became relatively slow with an increasing scanning rate, only the outer layer of the active electrode surface was used to store charge in the redox reaction process. Mathematical analysis revealed that the CV curve of the NCS@C-40 nanowire electrode material was higher than those of NCS@C-20 and NCS@C-80, suggesting a higher SC for the NCS@C electrode obtained with an electrodeposition time of 40 s.

GCD measurements were performed to understand the influence of the deposition time on the electrochemical characteristics of the NCS@C electrode. The SC was computed from the GCD curve using Eq. (5)^3^:

5 $$ C = \frac{{\left( {I \times t} \right)}}{{\left( {m \times \Delta V} \right)}} $$

where, ∆*V*, *t*, and *I* refer to the discharge potential range, discharge time (s), and discharge current (A), respectively, and *m* is the weight of the electroactive material on the working electrode (g). Figure 6a–d show the GCD measurement results of the NCS@C electrode materials prepared in the range of 0–0.55 V at various current densities (2–40 A g^−1^) in a 2 M KOH aqueous solution. Nonlinear charge–discharge curves, which were close to the level of the discharge platform, were observed. This is because the quasi-reversible redox reaction at the electrode/electrolyte interface induces pseudocapacitance behavior in the material. The symmetric GCD curves demonstrate that the material possessed excellent charge/discharge characteristics and electrochemical reversibility.

**Figure 7:**
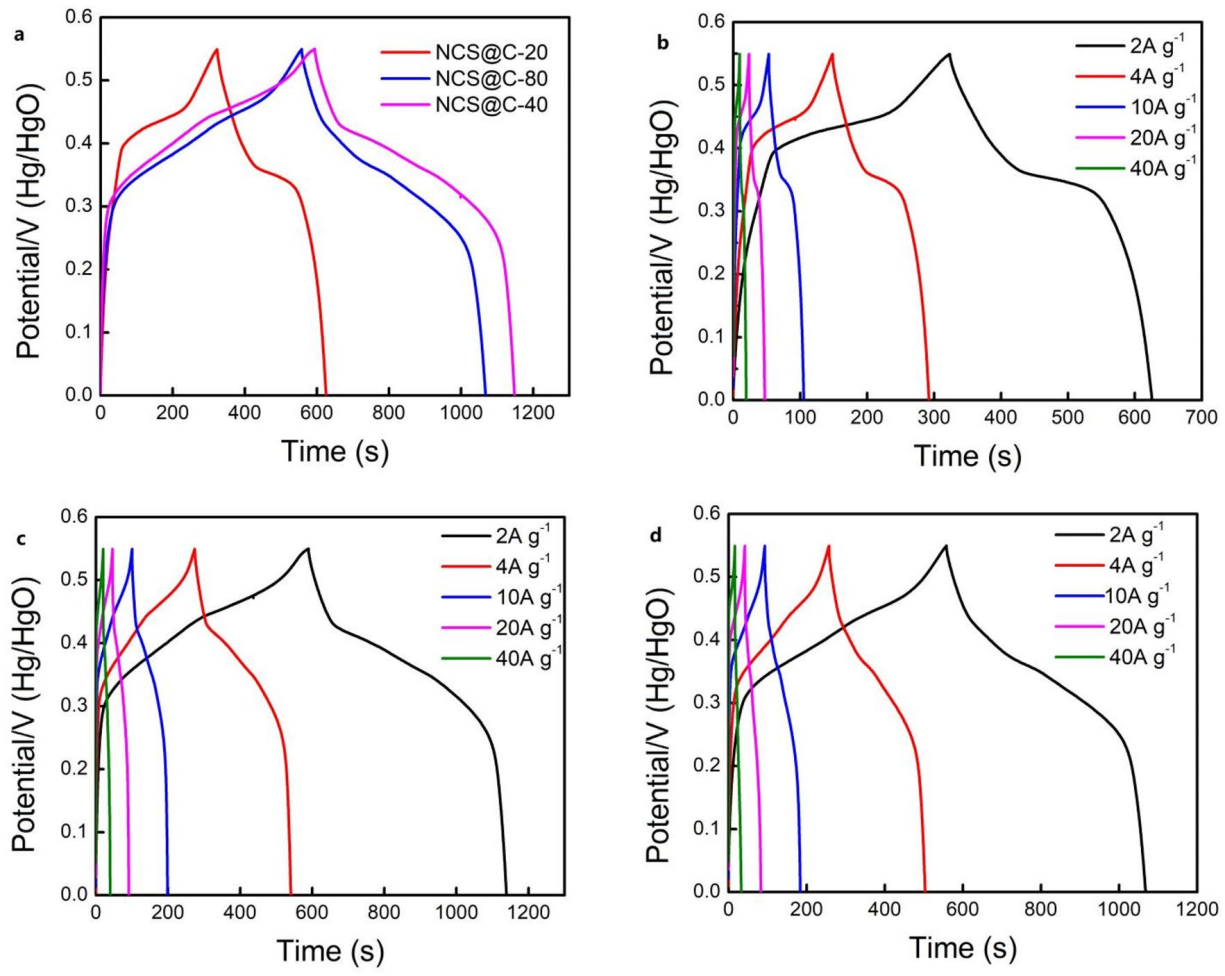
(**a**) Charge/discharge curves at a current density of 2 A g^−1^. Charge/discharge curves at different current densities of 2, 4, 10, 20, and 40 A g^−1^ for (**b**) NCS@C-20, (c) NCS@C-40, and (**d**) NCS@C-80.

Figure 7a shows that, at a current density of 2 A g^−1^, the electrochemical properties of the NCS@C electrode were tuned by adjusting the electrodeposition time. The NCS@C-20 electrode with an electrodeposition time of 20 s exhibited a specific capacity of 183 mAh g^−1^ (Fig. 7b). The NCS@C electrode material with an electrodeposition time of 40 s exhibited the highest SC (334.7 mAh g^−1^) (Fig. 7c). When the deposition time was extended to 80 s, the SC of NCS@C (310.3 mAh g^−1^) exhibited a decreasing trend (Fig. 7d). Therefore, the GCD curves of NCS@C exhibited a higher SC when the electrodeposition time was 40 s. Based on these findings, the advantages of the NCS nanosheet with an electrodeposition time of 40 s for enhancing the capacitance are clear.

**Figure 8:**
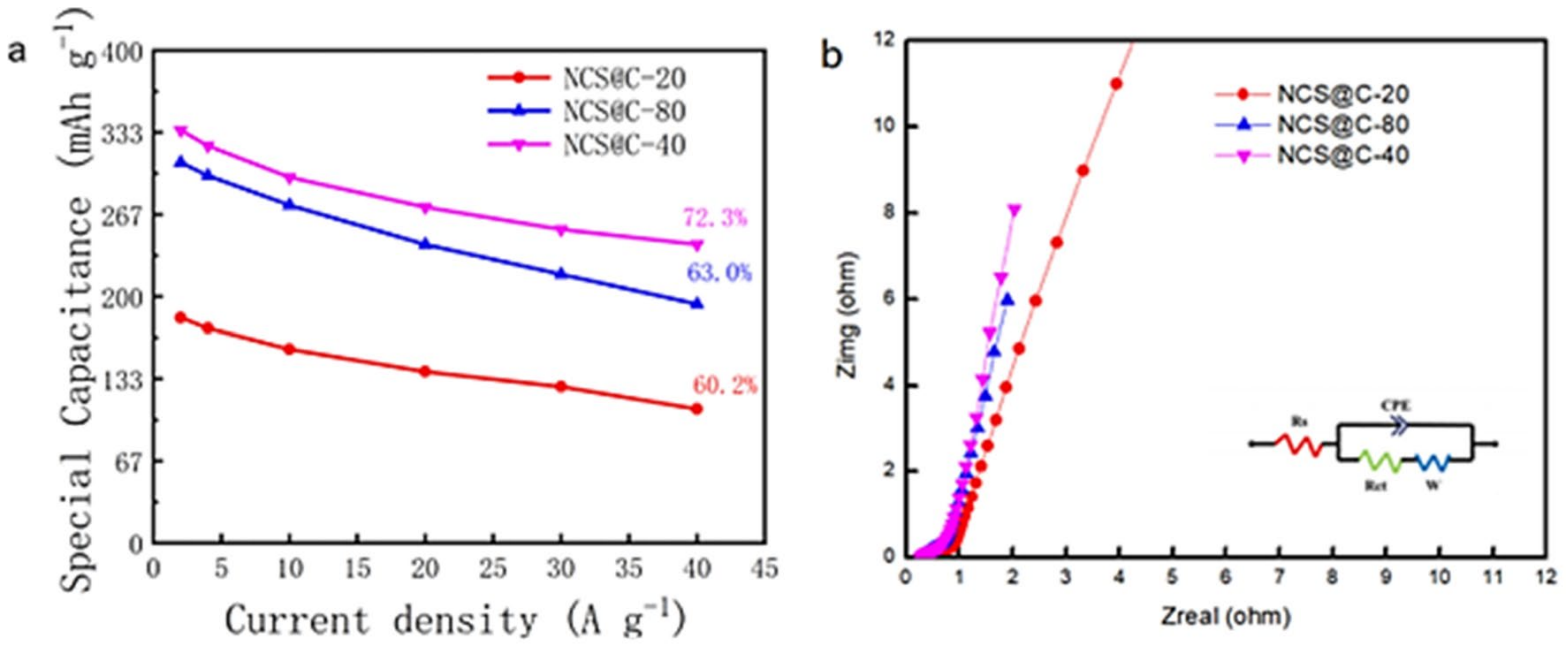
(**a**) SC of each sample at the current densities of 2, 4, 10, 20, 30, and 40 A g^−1^ and (b) Nyquist impedance plots of NCS@C.

According to Fig. 8a, NCS@C-40 exhibited the highest electrochemical property at the current densities of 2–40 A g^−1^. NCS@C-40 outperformed NCS@C-20 by approximately three-fold based on the SC at the same current density. A 72.3% SC was retained at a high current density of up to 2 A g^−1^, as compared to that with 40 A g^−1^, demonstrating the excellent rate capability of the material. The improvement in the NCS@C-40 sample rate capability and SC mainly benefited from its architecture with hierarchical pores, which enabled adequate exposure of the pseudocapacitance-active components. Moreover, the chemical binding between the NCS and carbon fiber promoted charge transportation between the current collector and the active components, which was conducive to a superior rate capability.

The fundamental behavior of the electrodes was examined using EIS analysis (Fig. 8b), which was performed in the frequency range of 100 kHz–0.01 Hz. The spectra of the two samples exhibited one arc in the high-frequency region and one inclined line in the low-frequency region^39,40^. The intercept of the plot at the solid line (Z') was consistent with the internal resistance in the high-frequency region (R_s_), representing the contact resistance at the electrolyte-active material and active material-collector interfaces. By fitting the low-frequency region, the slope of the curve was used to calculate the Warburg impedance (W), which indicates how the electrolyte diffuses through the pores of the electrode and protons in the material^41^. The EIS results showed that NCS possessed a lower R_s_ (0.23 Ω) and larger slope (W), demonstrating that NCS is an excellent base material. The NCS@C nanowire electrodes exhibited approximately the same impedance as the other samples (0.24 Ω) but the largest slope, indicating that the ion diffusion rate and conductivity were improved at the electrode/electrolyte interface.

**Figure 9:**
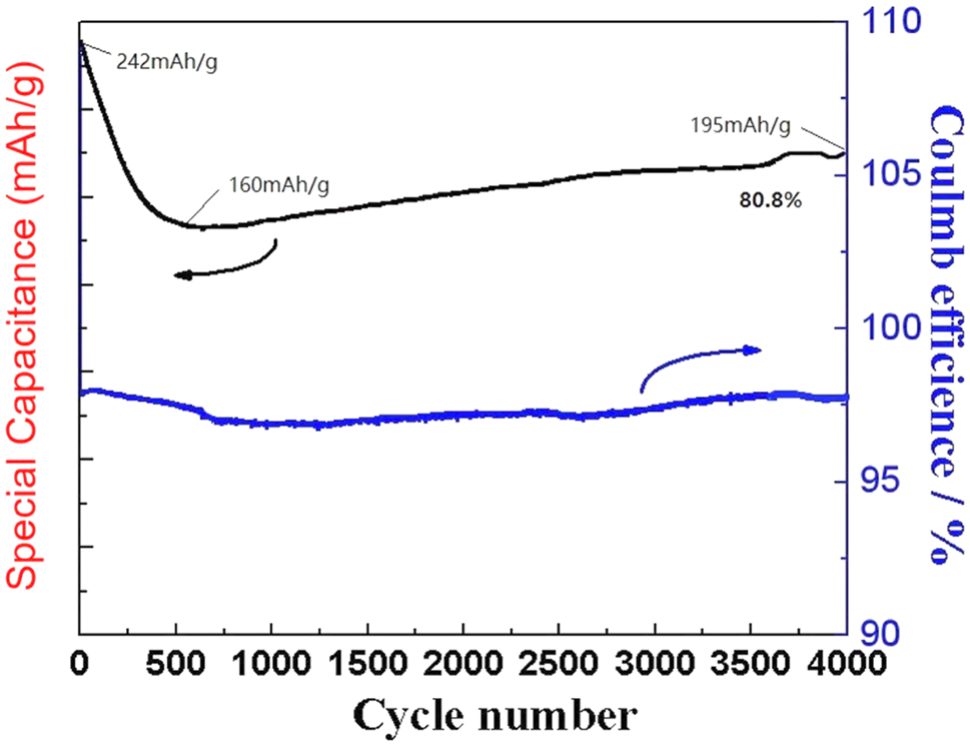
Cycling performance of the NCS@C nanotube arrays (4000 cycles) at a large current density of 40 A g^−1^.

Cycle performance tests were conducted as a key measurement index to evaluate the capability of the supercapacitors in real applications. Figure 8 shows the cycling durability of the specimens measured using a consecutive charge–discharge process at 40 A g^−1^. Based on these results, the NCS@C electrodes were expected to be in a state of inactivation, as confirmed by the cycling test. The specific capacity value decreased from 242 to 160 mAhg^−1^ at 500 cycles, then increased slowly and remained at approximately 195.53 mAhg^−1^ at the 4000th cycle. The initial increase in the specific capacity of the device was due to the activation of the electrodes through the exposure of more active sites in the hierarchical porous NCS and carbon nanofiber materials during cycling. As shown in Fig. 9, at a low current density, some side reactions occur during the electrochemical redox reaction, leading to an incomplete discharge. The charge and discharge times decreased with an increasing current density. The electrochemical process was primarily affected by the electric double layer; thus, the Coulombic efficiency increased. The NCS@C-40 electrode demonstrated exceptional cycling durability, maintaining 80.8% of the original capacity after 4000 cycles, suggesting that the complex and porous nanostructures and carbon layer can increase the cycling stability.

**Figure 10:**
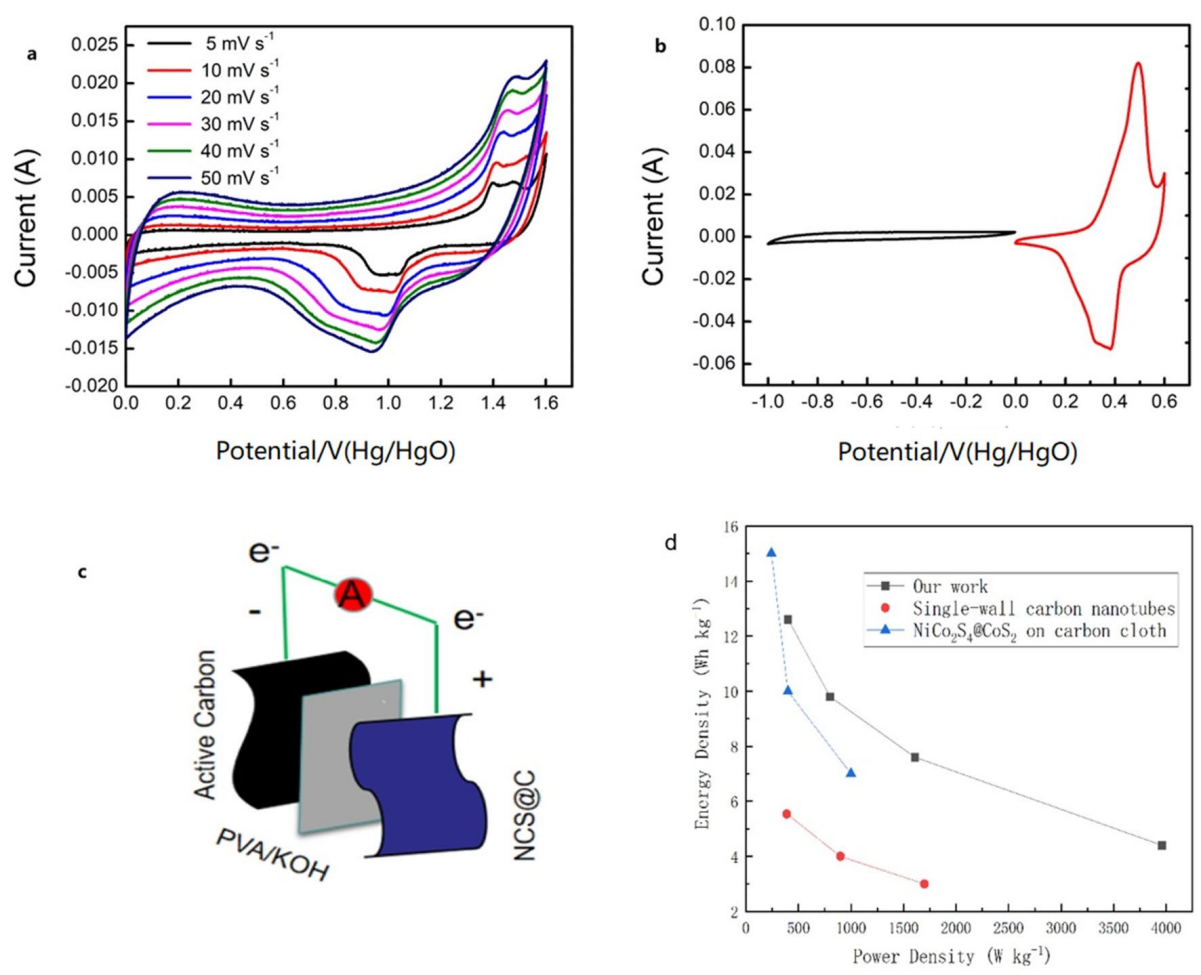
(**a**) CV curves of the device at different scan rates, (**b**) CV curves of the AC and NCS@C electrodes in the three-electrode system at a scan rate of 5 mV s^−1^, (**c**) schematic diagram of the ASC device, and (**d**) Ragone plot of the energy and power densities of the ASC device.

To further evaluate the potential applications of the NCS@C nanowires, an ASC test was performed with the NCS@C nanowires as the cathode and AC on the NF (denoted as NCS@C//AC) as the anode in a 6 M KOH aqueous electrolyte solution (Fig. 10).

The CV scans of the NCS@C//AC ASC at different scanning rates over a voltage range of 0–1.6 V are shown in Fig. 10b. The shapes of the CV curves of the NCS@C//AC ASC and NCS@C nanowire electrodes were similar in the three-electrode system, which is in agreement with the charge storage mechanism of the NCS@C nanowire electrode. The ASC device exhibited identical CV curves at different scanning rates from 5 to 50 mV s^−1^, showing that this material has appropriate rapid charging and discharging characteristics and displays Faraday pseudocapacitance characteristics as an electric double-layer capacitor. Moreover, a GCD test was used to characterize the electrochemical performance of the ASC devices at various current densities. When the current densities of the ASC were 0.5, 1, 2, and 5 A g^−1^, the corresponding SCs were 15.69, 12.22, 9.44, and 5.55 mAh g^−1^, respectively. Both the charging and discharging curves were highly symmetric, indicating that the ASC exhibited good electrochemical reversibility.

The power and energy densities are two critical parameters for evaluating the performance of the electrode material of the ASC in practical applications, and can be obtained using Eqs. (6) and (7):

6 $$ E = I \times \int {Vdt/m \times 3.6} $$

7 $$ P = E \times 3600/t $$

where *E* and *P* refer to the energy density (Wh kg^−1^) and power density (W kg^−1^), respectively,* C* is the SC, *V* is the potential range, and *t* is the discharging time. A high energy density of 12.91 Wh kg^−1^, corresponding to a power density of 358 W kg^−1^, was achieved using the ASC at a current density of 0.5 A g^−1^ (Fig. 9d). These results suggest that the NCS@C nanowires can be used as positive electrodes in ASC devices to obtain high energy density, which is required for the practical application of flexible supercapacitors.

## Conclusions

NCS nanowires were grown on carbon fibers as binder-free electrodes for supercapacitors by combining a hydrothermal treatment and electrochemical deposition. NCS nanowires with a reactive surface area were evenly distributed on the surface of the carbon fiber to achieve rapid transport of electrons and improve the electrochemical performance. At a current density of 2 A g^−1^, the NCS@C-40 exhibited a high SC of up to 334.7mAh g^−1^. At a high current density (40 A g^−1^), the SC of NCS@C was 242.0 mAh g^−1^, corresponding to a 72.3% retention rate. The SC of NCS@C remained at 80.8% after 4,000 cycles. A high energy density of 12.91 Wh kg^−1^ and a power density of 358 W kg^−1^ were achieved by the ASC at a current density of 0.5 A g^−1^. These findings strongly suggest that the NCS@C nanowire is a competitive candidate as a positive electrode for ASC devices to obtain high energy density, which is required for the practical application of flexible supercapacitors (Supplementary File 1).

### Supplementary Information

Supplementary Information.

## Data Availability

All data generated or analysed during this study are included in this published article [and its supplementary information files].
