# Supplementary figures and images for: A flexible wearable self-supporting hybrid supercapacitor device based on hierarchical nickel cobalt sulfide@C electrode
Source: Sci Rep. 2023 Sep 20;13:15555. doi: 10.1038/s41598-023-42278-9 (PMC10511439; doi:10.1038/s41598-023-42278-9)

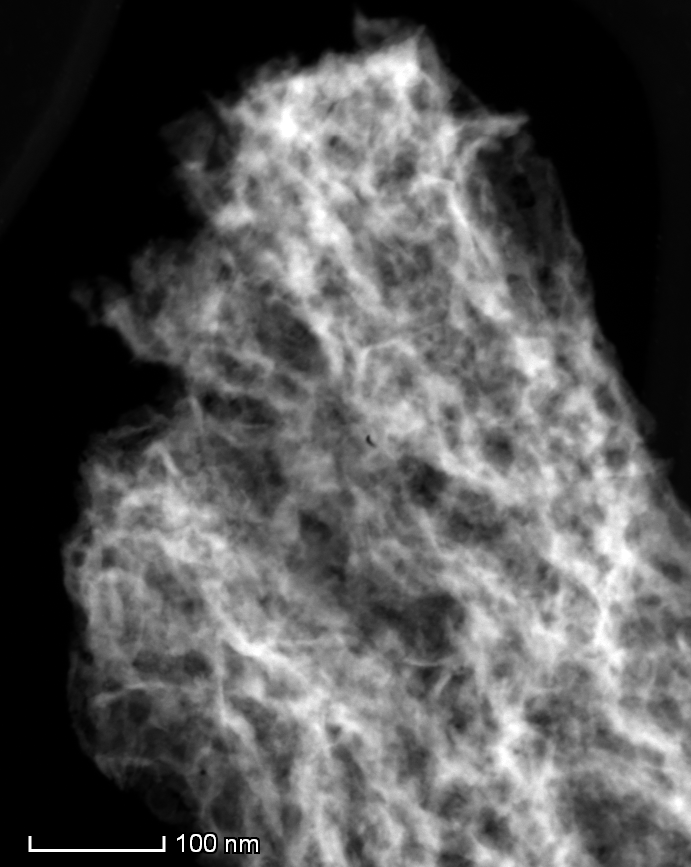

Supplement: Supplementary file 1 — Supplementary Information. [file 41598_2023_42278_MOESM1_ESM.zip › Raw Data/EDS-TEM-SAED/EDS/SI EDS-HAADF 1511-1.tif]

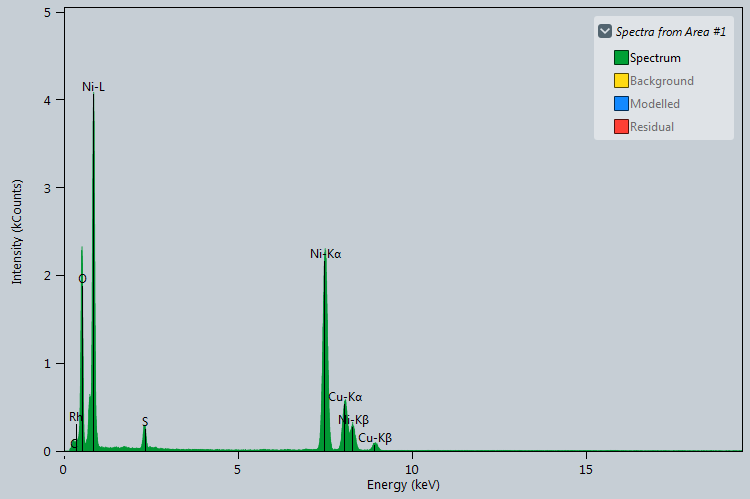

Supplement: Supplementary file 1 — Supplementary Information. [file 41598_2023_42278_MOESM1_ESM.zip › Raw Data/EDS-TEM-SAED/EDS/SI EDS-HAADF 1511.png]

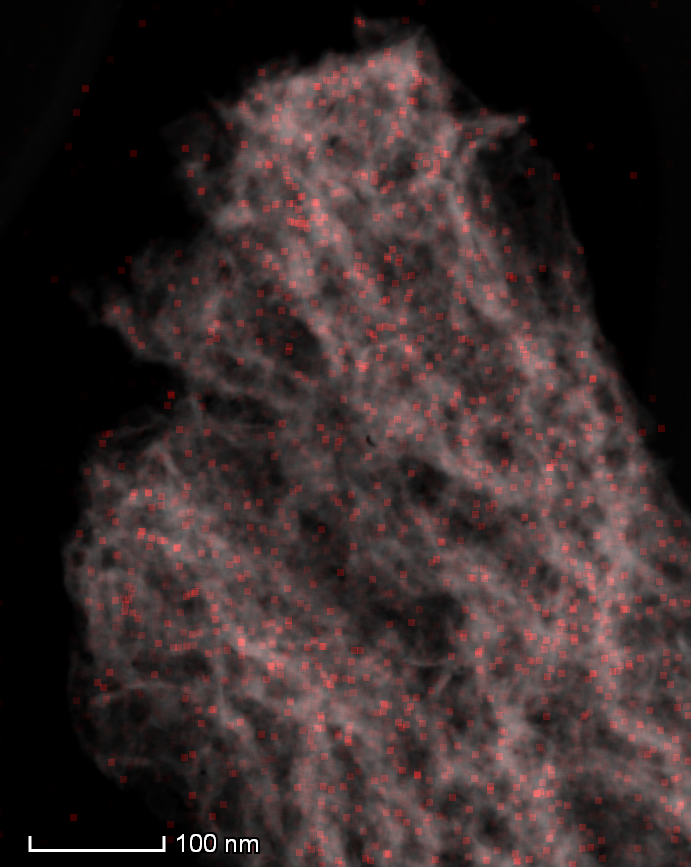

Supplement: Supplementary file 1 — Supplementary Information. [file 41598_2023_42278_MOESM1_ESM.zip › Raw Data/EDS-TEM-SAED/EDS/SI EDS-HAADF 1511Co.tif]

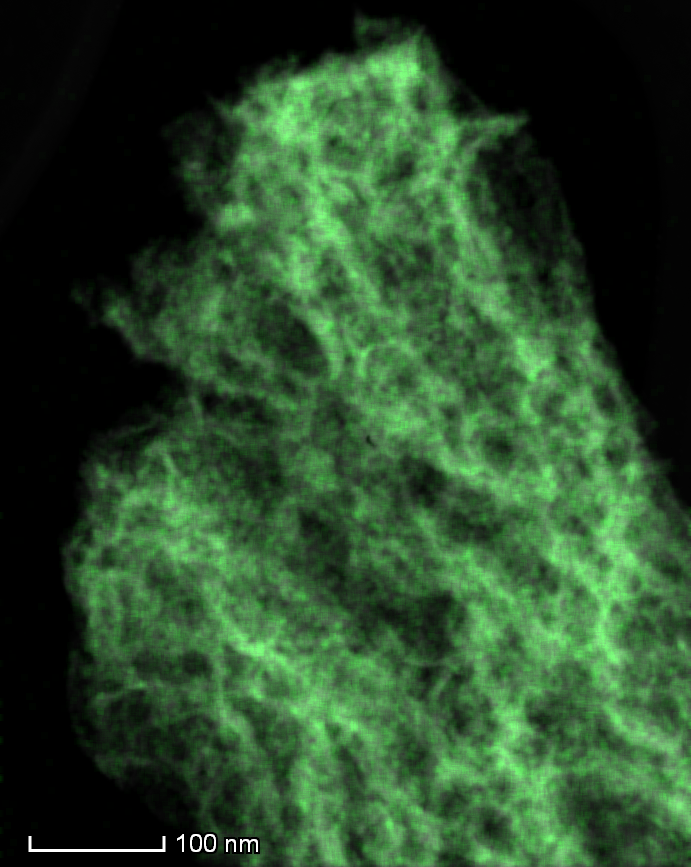

Supplement: Supplementary file 1 — Supplementary Information. [file 41598_2023_42278_MOESM1_ESM.zip › Raw Data/EDS-TEM-SAED/EDS/SI EDS-HAADF 1511Ni.tif]

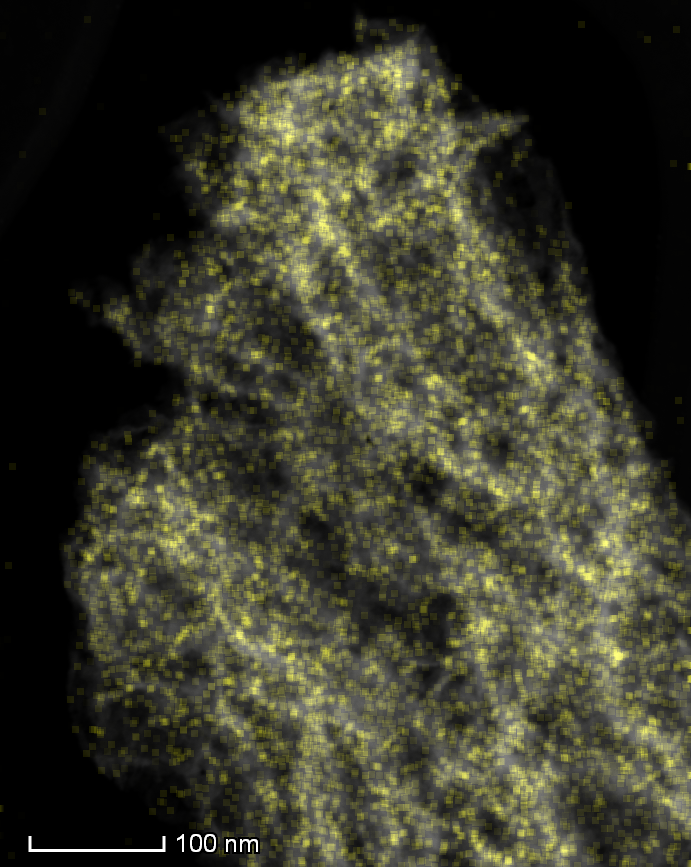

Supplement: Supplementary file 1 — Supplementary Information. [file 41598_2023_42278_MOESM1_ESM.zip › Raw Data/EDS-TEM-SAED/EDS/SI EDS-HAADF 1511S.tif]

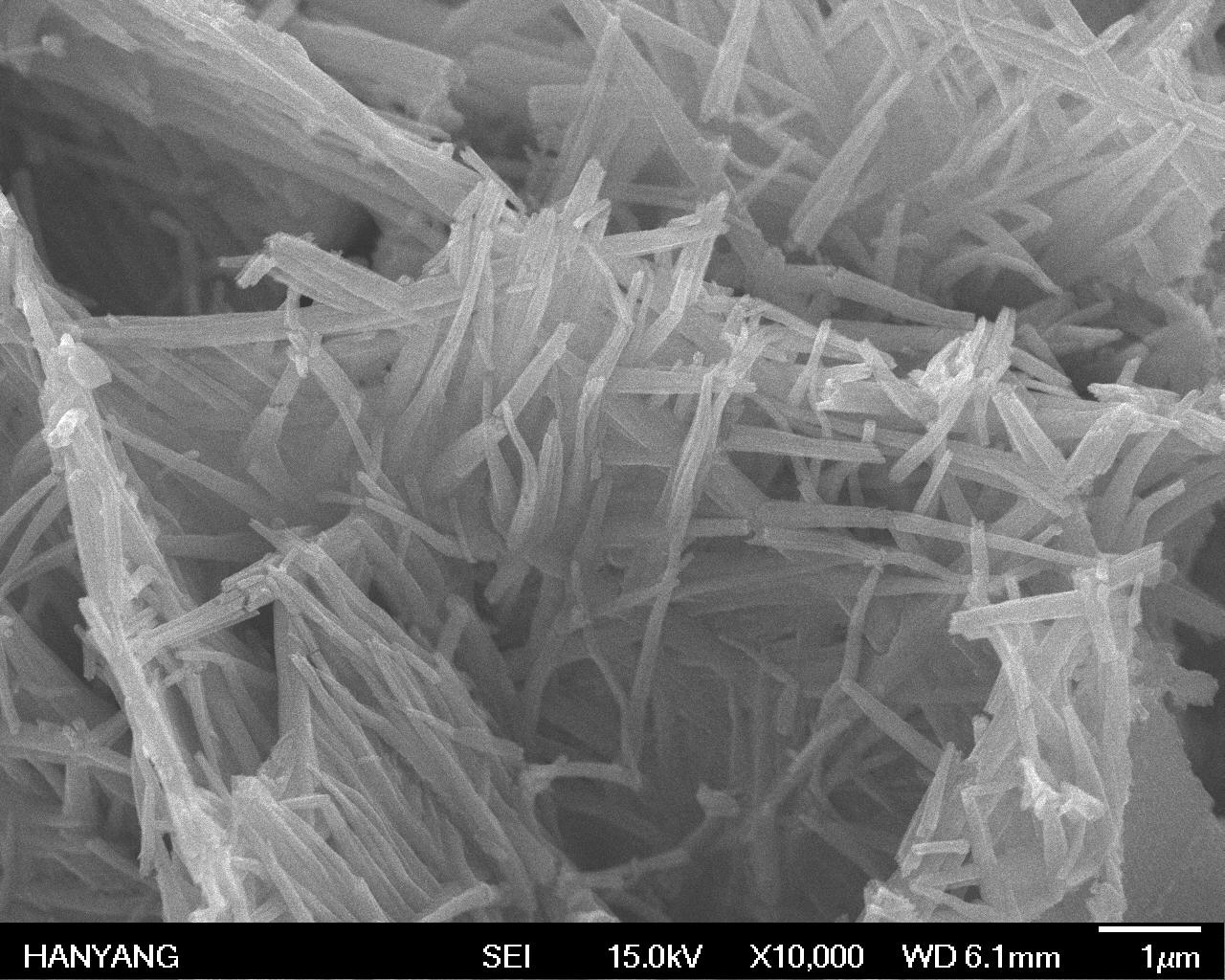

Supplement: Supplementary file 1 — Supplementary Information. [file 41598_2023_42278_MOESM1_ESM.zip › Raw Data/Fig.1/Fig.1a.jpg]

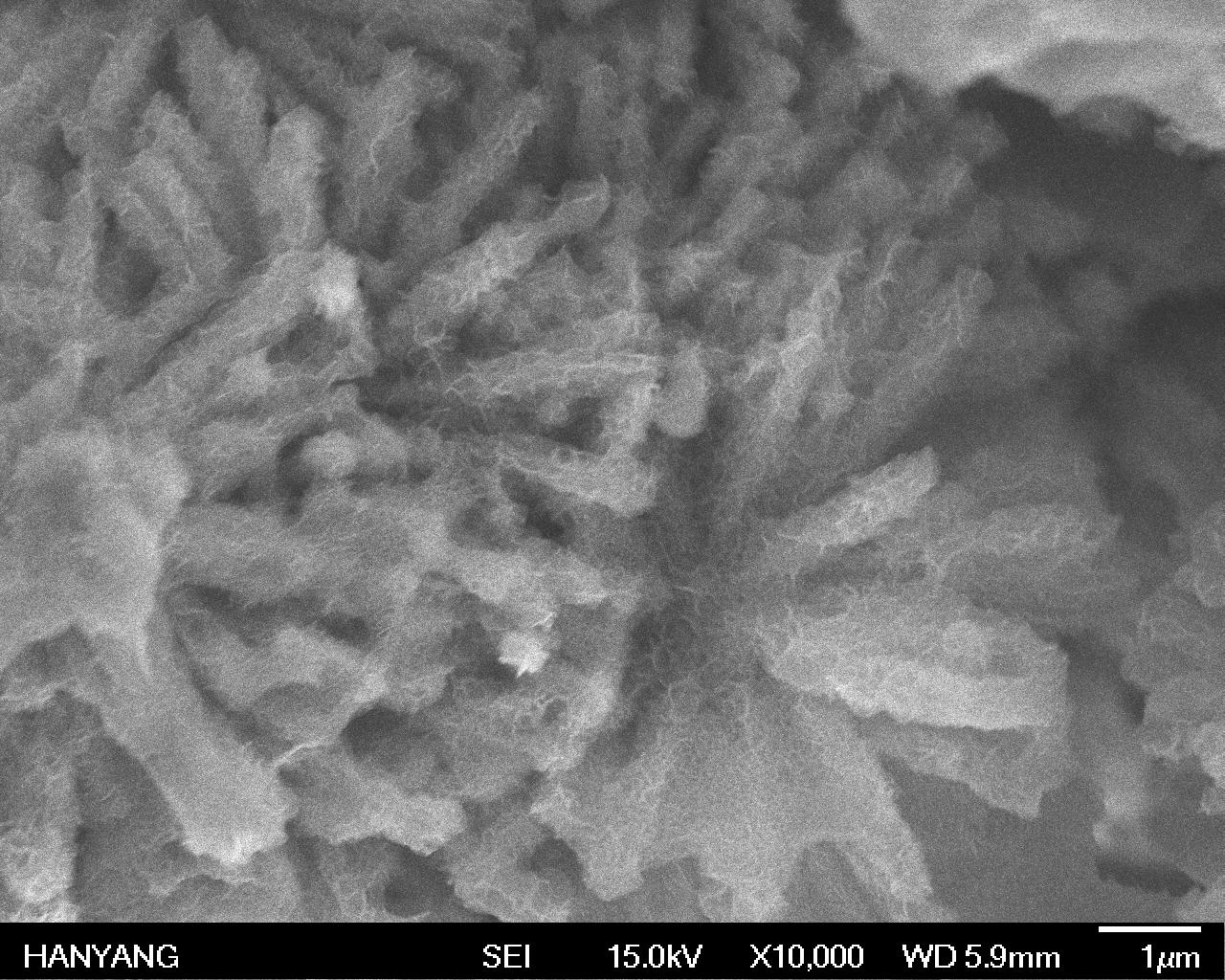

Supplement: Supplementary file 1 — Supplementary Information. [file 41598_2023_42278_MOESM1_ESM.zip › Raw Data/Fig.1/Fig.1c.jpg]

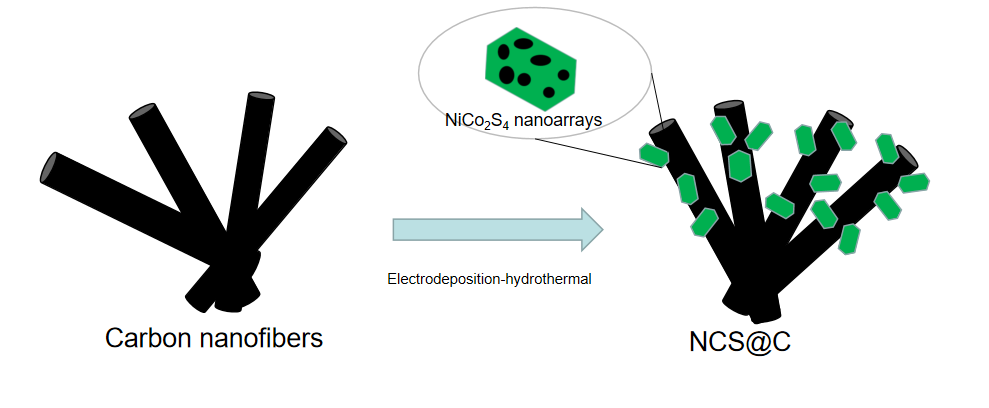

Supplement: Supplementary file 1 — Supplementary Information. [file 41598_2023_42278_MOESM1_ESM.zip › Raw Data/Fig.1/Fig.1e.png]

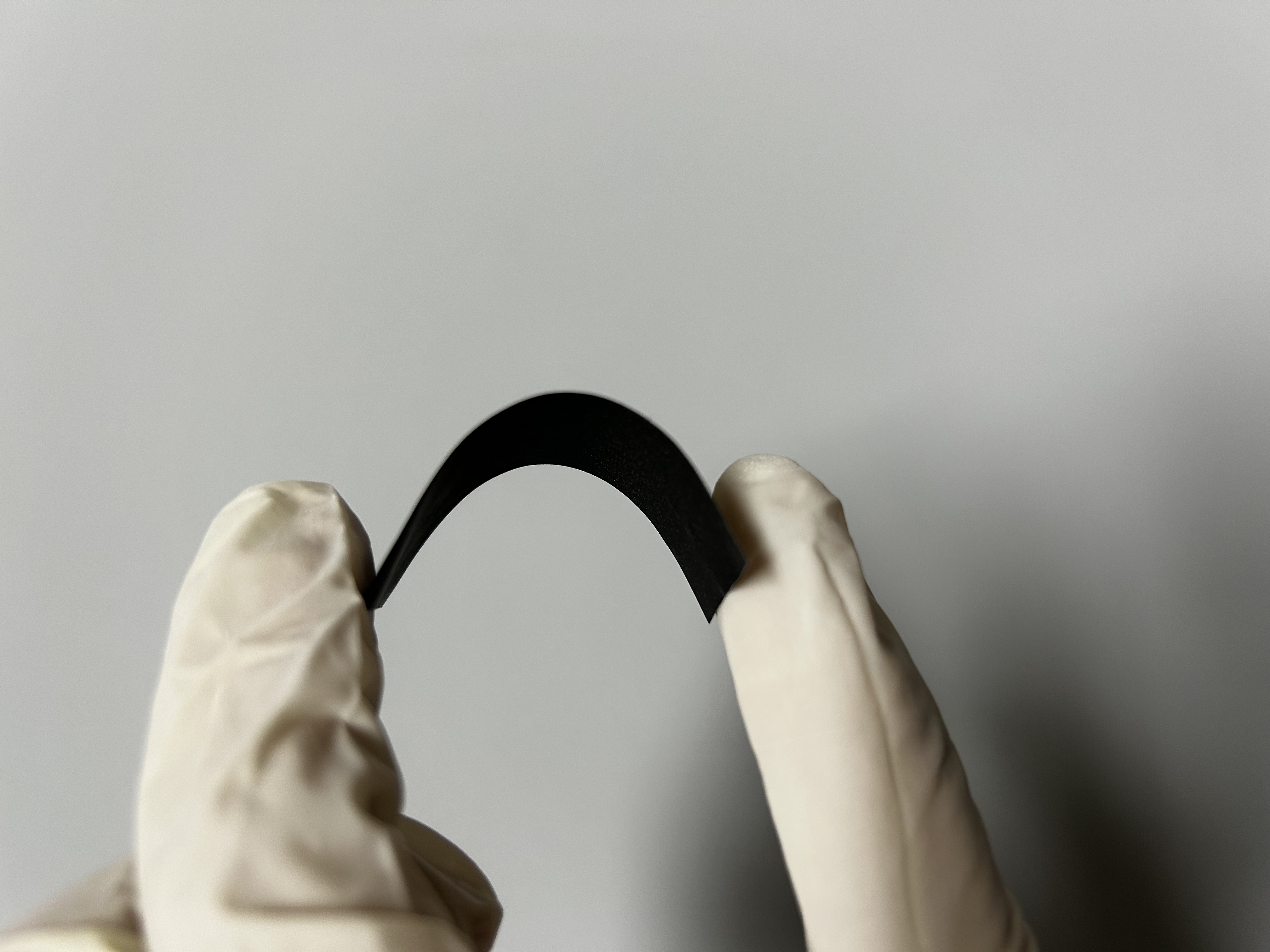

Supplement: Supplementary file 1 — Supplementary Information. [file 41598_2023_42278_MOESM1_ESM.zip › Raw Data/Fig.1/Fig1b.jpg]
